# A New Kinetic Spectrophotometric Method for the Quantitation of Amorolfine

**DOI:** 10.1155/2017/9812894

**Published:** 2017-02-28

**Authors:** César Soto, Cristian Poza, David Contreras, Jorge Yáñez, Fallon Nacaratte, M. Inés Toral

**Affiliations:** ^1^Departamento de Química Analítica e Inorgánica, Facultad de Ciencias Químicas, Universidad de Concepción, Concepción, Chile; ^2^Laboratorio de Química Analítica, Facultad de Ciencias, Universidad de Chile, Santiago, Chile

## Abstract

Amorolfine (AOF) is a compound with fungicide activity based on the dual inhibition of growth of the fungal cell membrane, the biosynthesis and accumulation of sterols, and the reduction of ergosterol. In this work a sensitive kinetic and spectrophotometric method for the AOF quantitation based on the AOF oxidation by means of KMnO_4_ at 30 min (fixed time), pH alkaline, and ionic strength controlled was developed. Measurements of changes in absorbance at 610 nm were used as criterion of the oxidation progress. In order to maximize the sensitivity, different experimental reaction parameters were carefully studied via factorial screening and optimized by multivariate method. The linearity, intraday, and interday assay precision and accuracy were determined. The absorbance-concentration plot corresponding to tap water spiked samples was rectilinear, over the range of 7.56 × 10^−6^–3.22 × 10^−5^ mol L^−1^, with detection and quantitation limits of 2.49 × 10^−6^ mol L^−1^ and 7.56 × 10^−6^ mol L^−1^, respectively. The proposed method was successfully validated for the application of the determination of the drug in the spiked tap water samples and the percentage recoveries were 94.0–105.0%. The method is simple and does not require expensive instruments or complicated extraction steps of the reaction product.

## 1. Introduction

**Figure 1:**
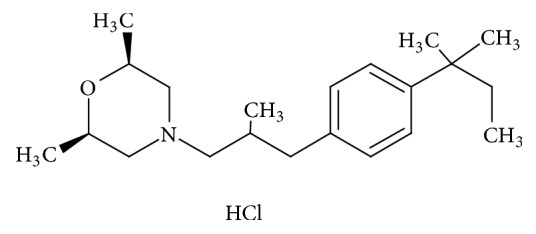
Amorolfine (AOF).

Fungi are widely distributed in nature and most of them are pathogens which are the major causes of morbidity and mortality in patients with compromised immune systems (cancer, polytrauma, and HIV) that present a high risk of infection by opportunistic organisms [1, 2]. In recent decades, therapeutic treatments for systemic fungal diseases have improved and expanded their use through more effective and less toxic novel drugs [3, 4]. New fungicides, such as amorolfine (AOF) or (2R, 6S)-rel-4-[3-[4-(1,1-dimethylpropyl)phenyl]-2-methylpropyl]-2,6-dimethyl-morpholine hydrochloride (Figure 1), have been incorporated into existing fungicides. This drug is a morpholine derivate [5] owning fungicide activity in a broad spectrum. This activity is based on the dual inhibition of growth of the fungal cell membrane and the action on the biosynthesis of sterols, producing their accumulation and reducing the amount of ergosterol [6, 7]. Topical presentation used for treatment of skin and nails is widely used [8]. A few methods for the quantitative determination of AOF in all types of samples (biological, environmental, and pharmaceutical) have been reported, including HPLC [9, 10] and spectrophotometric method [11]. In recent years kinetic spectrophotometric methods [12, 13] have been used for the determination of pharmaceutical compounds [14–17], in biological fluids [18], and natural and tap water [19–25]. These methods are suitable due to their characteristics of specificity and sensitivity [26], since absorbance variation in the time is measured in nonequilibrium condition [27, 28].

This work describes the development and validation of a kinetic and spectrophotometric method for quantitation in low concentration of AOF in tap water spiked samples. The AOF oxidation by means of KMnO_4_ was used and the reaction product K_2_MnO_4_ [29] was spectrophotometric determined at 610 nm.

## 2. Experimental

### 2.1. Instruments

A Perkin Elmer Lambda 35 double beams spectrophotometer (USA) with 10 mm quartz cells was used for measurements. For all solutions, the spectra were recorded on the range between 190 and 800 nm against blank, using sampling intervals of 0.2 nm with a scan speed of 480 nm min^−1^. The spectral data were processed by Perkin Elmer UV Win Lab Data Processor and Viewer 1.00. The solid samples were weighted with a ±0.01 mg of uncertainty using AS 60/220/C/2 analytical balance.

### 2.2. Reagents

AOF was purchased from Toronto Chemical Research, TCR® (Canada). All reagents were of analytical reagent grade and all solutions were prepared with Milli-Q water. Stock solutions of 1.11 × 10^−3^ mol L^−1^ of AOF were prepared by dissolving 10.0 mg and diluting 25 mL with deionized water in a volumetric flask. The same solution was used to prepare samples with different concentrations by appropriate dilution using deionized water. Aqueous solutions of NaOH 1.0 mol L^−1^, KMnO_4_ 9.8 × 10^−2^ mol L^−1^, and Na_2_SO_4_ 2.0 mol L^−1^ were prepared from Merck (Germany) reagents.

## 3. Procedures

### 3.1. Kinetic Procedure

Samples were prepared using accurately aliquots of AOF stock solutions that were transferred into 5 mL volumetric flasks. Proper amounts of KMnO_4_ 9.8 × 10^−2^ mol L^−1^, NaOH 1.0 mol L^−1^, and Na_2_SO_4_ 2.0 mol L^−1^ were added to achieve the oxidation reaction in optimal conditions. The samples were heated in water bath at 50 ± 0.1°C for 30 min and the absorbance of solutions was measured at 610 nm. External calibration using prepared standards of AOF and blank in the range 6.0 × 10^−6^ to 5.0 × 10^−5^ mol L^−1^ was carried out. Thereafter, the corresponding regression equation is attained. Furthermore, log⁡*v* v/s log⁡[AOF] was plotted to get the order of the reaction.

### 3.2. Factorial Optimization Parameters

In order to optimize the reaction, an experimental design with statistical software (Modde 7) was used, considering a factorial design 2^4^ using the factors on Table 1. Then a circumscribed central composite design (CCC) was performed, using the factors NaOH concentration (0.18, 0.26, and 0.34 mol L^−1^) and temperature (50, 65, and 80°C) and maintaining KMnO_4_ 9.0 × 10^−4^ mol L^−1^, Na_2_SO_4_ 0.2 mol L^−1^. For optimization AOF concentration was 2.3 × 10^−5^ mol L^−1^ and the response used was the absorbance at 610 nm after 30 min of reaction time.

### 3.3. Procedure for Determination of AOF in Tap Water Spiked Samples

The developed method was applied for the quantitation of AOF in spiked tap water samples. The samples analyzed (500 mL) were gathered from taps located in two labs. The samples were collected in polyethylene bottles without adding any preservative agent and analyzed within 5 h. In order to remove the suspended fine particles (organic and inorganic matter) and dissolved gases, which are considered as potential interferences that can be oxidized by KMnO_4_, the samples were boiled (5 min) and filtered (0.25 *μ*m). Aliquots of the tap water were spiked with known concentrations of AOF and then the samples were prepared according to the procedure described above.

## 4. Results and Disscusions

Permanganate in alkaline medium was used in the development of a kinetic method for the determination of AOF. The oxidation of this drug under specific conditions of pH and ionic strength causes color changes to bluish green corresponding to the manganate K_2_MnO_4_ (the main reaction product) producing new absorption bands at 450 and 610 nm. The intensity of the color increases with the concentration of this reaction product. The influences of the following variables, reaction time (*t*), temperature (*T*°), concentrations of NaOH (pH), KMnO_4_, and Na_2_SO_4_ (effect of ionic strength), were studied by the univariate method. Then only the variables involved in the formation of K_2_MnO_4_ were optimized. The reaction was monitored at 610 nm using AOF 2.2 × 10^−5^ mol L^−1^.

### 4.1. Variables Studies

**Figure 2:**
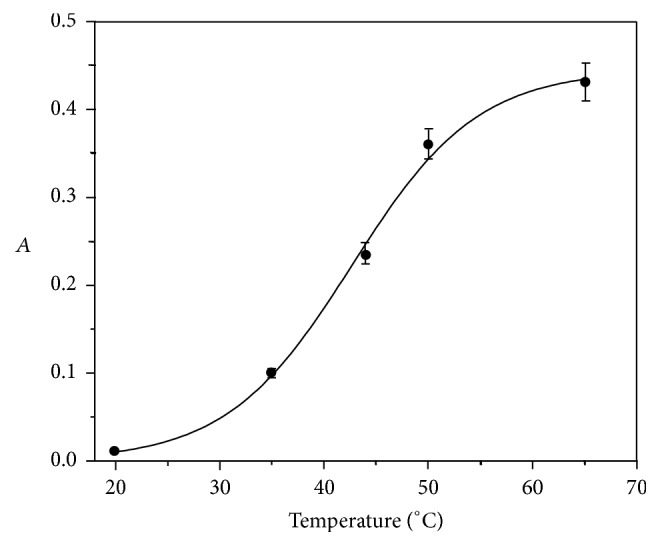
Effect of the temperature on the system AOF-KMnO_4_ (2.3 × 10^−5^ and 9.0 × 10^−4^ mol L^−1^, resp.) with Na_2_SO_4_ 0.20 mol L^−1^, NaOH 0.30 mol L^−1^, monitoring at 610 nm, and* t* = 30 min.

The effect of the temperature on the reaction rate over the range 20 to 65°C was studied (Figure 2). The oxidation of AOF is favored by increasing temperature but from 50°C this effect decreases. This can be attributed to the permanganate self-decomposition over 65°C. Therefore, the temperature was adjusted at 50°C.

**Figure 3:**
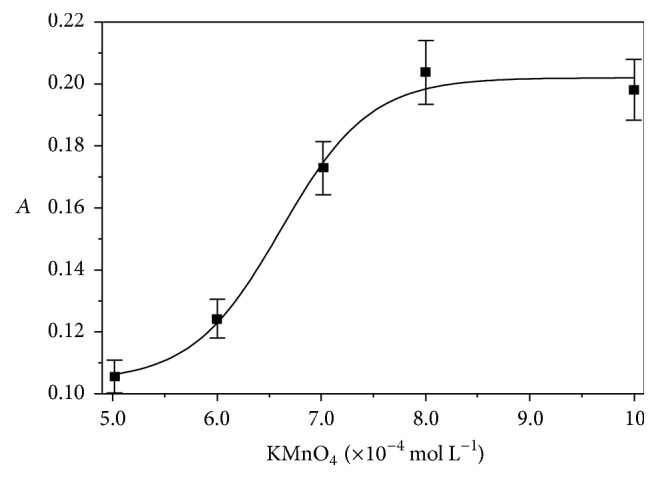
Concentration effect of KMnO_4_ (5.0 × 10^−4^–1.0 × 10^−3^ mol L^−1^) on the AOF-KMnO_4_ system, with AOF 2.2 × 10^−5^ mol L^−1^, Na_2_SO_4_ 0.20 mol L^−1^, NaOH 0.30 mol L^−1^,* T*° = 50°C,* t* = 30 min, and monitoring at 610 nm.

The effect of KMnO_4_ concentration was studied over the range 5.0 × 10^−4^–9.1 × 10^−4^ mol L^−1^ (Figure 3). The results show that reaction rate increased as oxidant concentration augmented, evidencing a proportional dependence. Up to 8.0 × 10^−4^ mol L^−1^ KMnO_4_ does not affect the reaction rate which depends on substrate concentration. This value was initially selected.

**Figure 4:**
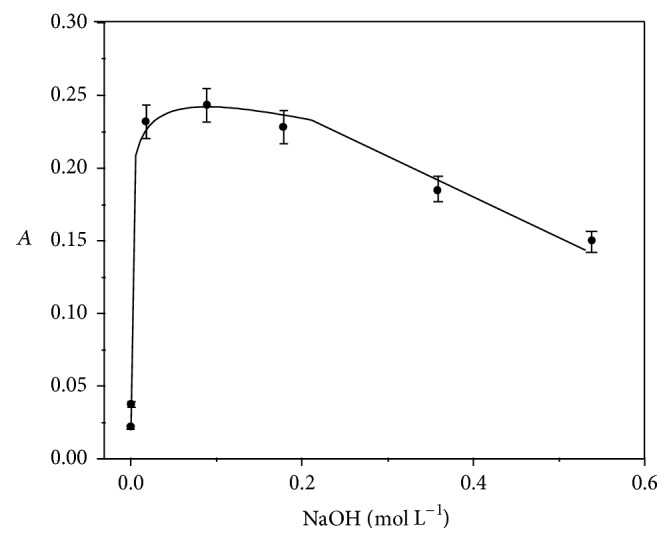
Concentration effect of NaOH (4.0 × 10^−4^–0.56 mol L^−1^) on the AOF-KMnO_4_ system, with AOF 2.3 × 10^−5^ mol L^−1^, KMnO_4_ 9.0 × 10^−4^ mol L^−1^, Na_2_SO_4_ 0.20 mol L^−1^,* t* = 30 min,* T*° = 50°C, and monitoring at 610 nm.

The AOF oxidation with KMnO_4_ takes place in alkaline medium. The study of the medium effect (pH) was carried out with different NaOH concentrations ranging from 4.0 × 10^−4^ to 0.56 mol L^−1^ (Figure 4). Experimentally the stability of K_2_MnO_4_ (reduction product) occurred in the range of 0.018 and 0.34 mol L^−1^.

**Figure 5:**
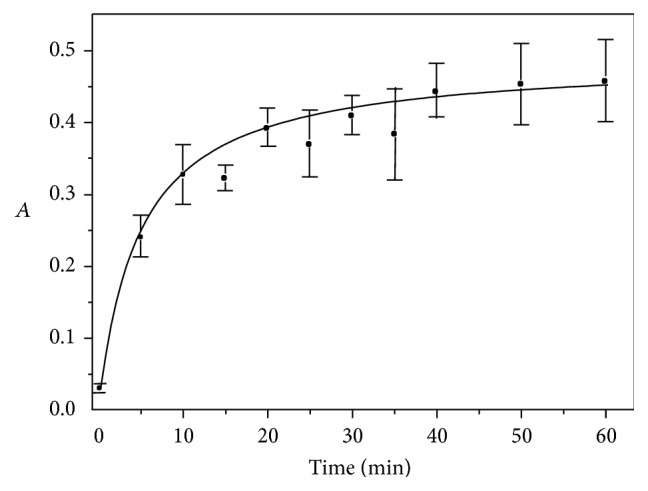
Effect of reaction time (*t*) on the AOF-KMnO_4_ system, at NaOH 0.34 mol L^−1^, AOF 2.3 × 10^−5^ mol L^−1^, KMnO_4_ 9.0 × 10^−4^ mol L^−1^, NaOH 0.30 mol L^−1^, Na_2_SO_4_ 0.20 mol L^−1^,* T*° = 50°C, and monitoring at 610 nm.

The effect of time of reaction (*t*) was also studied by measuring the absorbance at the 0–60 min interval (Figure 5). The absorbance values increased proportionally up to 30 min; longer time periods present negligible effects in absorbance values. Hence, 30 min of reaction time was selected.

**Figure 6:**
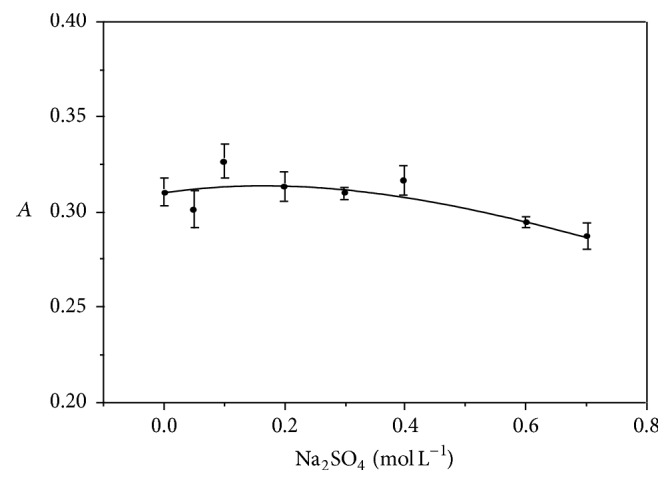
Concentration effect of Na_2_SO_4_ (0–0.7 mol L^−1^) on the AOF-KMnO_4_ system, with AOF 2.3 × 10^−5^ mol L^−1^, NaOH 0.30 mol L^−1^, KMnO_4_ 9.0 × 10^−4^ mol L^−1^,* t* = 30 min,* T*° = 50°C, and monitoring at 610 nm.

Additionally, the effect of Na_2_SO_4_ concentration (ionic strength), on the reaction rate, was studied (Figure 6). Since the variation of this concentration did not affect AOF oxidation, 0.2 mol L^−1^ was used to maintain constant ionic strength.

### 4.2. Variables Optimization

**Figure 7:**
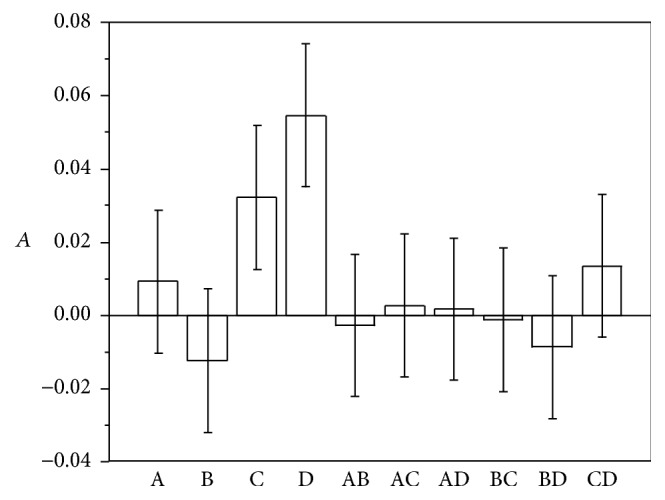
Influence of variables on the AOF-KMnO_4_ system for the factorial design (A = KMnO_4_; B = Na_2_SO_4_; C = NaOH; and D =* T*°).

**Figure 8:**
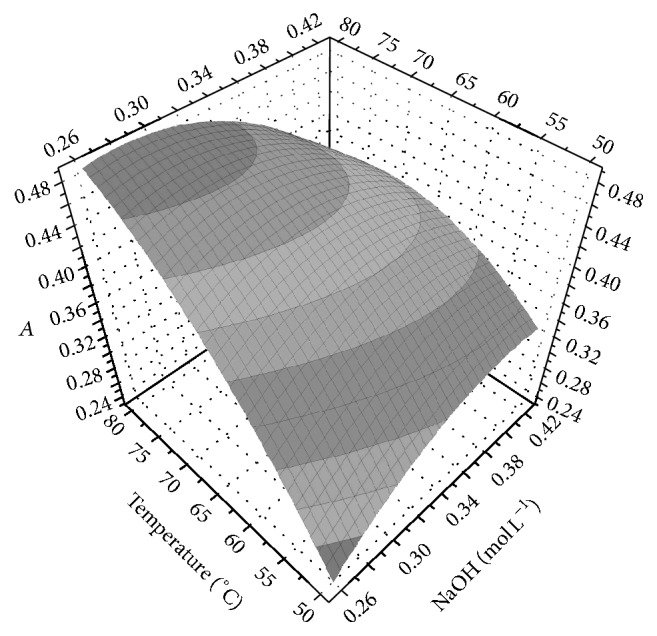
Graphic response surface of the circumscribing central composite design (CCC) for the AOF-KMnO_4_ system.

Considering the above results, a factorial design 2^4^ was performed, using the factors presented in Table 1. The* t* and AOF concentration values used were 30 min and 2.3 × 10^−5^ mol L^−1^, respectively. The results showed that the reaction is influenced mainly by the temperature and NaOH concentration (Figure 7). In order to optimize the reaction a circumscribing central composite design (CCC) was applied considering these 2 variables using the values indicated on Section 3.2 and constant values for KMnO_4_ = 9.0 × 10^−4^ mol L^−1^, Na_2_SO_4_ = 0.2 mol L^−1^, and* t* = 30 min. The optimal values obtained from the model were temperature 86°C and NaOH 0.26 mol L^−1^ (Figure 8). Temperature values above 65°C cause the precipitation of MnO_2_; then the experimental work is achievable at a lower temperature, where 50°C was selected. Using this value the model maximizes the response signal with NaOH 0.34 mol L^−1^. In the experimental verification, the values predicted by the model were obtained.

### 4.3. Stoichiometry Determination

**Scheme 1:**
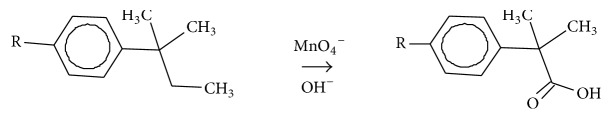
Reaction between AOF and KMnO_4_ in alkaline medium.

The stoichiometry of the reaction was studied using optimized experimental conditions, monitoring at 610 nm and the limit logarithmic method [30]. A plot of log⁡*A* v/s log⁡[AOF] at a constant KMnO_4_ concentration gave a straight line with a slope of 0.723. A plot of log⁡*A* v/s log⁡[KMnO_4_] at a constant AOF concentration gave a straight line with a slope of 0.685. Thus, the molar ratio of the reaction is 0.723 : 0.685 ≈ 1 : 1. Based on the obtained molar relation and other similar reactions described [29], the alkyl group (1,1-dimethylpropyl in our case) attached to the aromatic ring was oxidized more easily than the other groups. The propyl group is oxidized by KMnO_4_ in alkaline conditions turning into the corresponding carboxilic acid in which the permanganate is reduced to the manganate (colored species). The proposed reaction pathway is shown in Scheme 1.

### 4.4. Kinetic of the Reaction

Considering the pseudo-first order reaction conditions ([KMnO_4_] > [AOF]) and the optimized variables, the kinetic behavior of reaction can be represented by the equation* v* = *k*′[AOF]^*n*^, with the plot of log⁡*v* v/s log⁡[AOF], the order of reaction (*n*) and rate constant (*k*′) were obtained, and the values were 0.938 (≈1) and 4.70 s^−1^, respectively.

### 4.5. Evaluation of the Kinetic Methods

The pseudo-first order reaction rate (*v* = 4.70 s^−1^[AOF]^0.938^) was the basis for several experiments conducted to obtain AOF concentration. The kinetic methods of initial rate, constant rate, and fixed time were tried and selected based on the applicability, sensitivity, intercept, and* R*^*2*^. The first two methods were discarded, because they presented low linearity, reproducibility, and sensitivity. It was concluded that the fixed time method presents linear correlation for each value of time studied.

*Initial Rate*. The curves of absorbance (at 610 nm) v/s* t* (s) are obtained for AOF concentrations over the range 6.0 × 10^−6^ to 5.0 × 10^−5^ mol L^−1^ of AOF. Then, tangents were drawn for each curve with *t* = 250 s. Afterwards, the respective slopes (*K*′′) were obtained. Equation (1) was obtained from the graph of *K*′′ v/s [AOF] and it corresponds to a kinetic behavior of pseudo-zero order (no determining step of the reaction). *R*^2^ and slope values indicate low linearity and sensitivity, respectively. For this reason this method was discarded.

(1) K″=7.33C+2.51×10−5,R2=0.8749.

*Constant Rate*. For this method, the plots of log⁡*A* (at 610 nm) v/s *t* (0–1800 s) for range of AOF concentration of 6.0 × 10^−6^ to 5.0 × 10^−5^ mol L^−1^ were performed, obtaining straight lines, with slope = *K*′/2.303 (pseudo-first order). Then, the drug concentration v/s *K*′ was plotted (2). Although this method produces a better linear correlation with respect to the initial rate, the low slope makes it impossible to obtain the necessary parameters to develop an analytical method.

(2) K′=0.720C−4.40×10−4,R2=0.9431.

*Fixed Time*. In this method a preselected group of fixed time values was accurately measured (0 to 60 min). For each one, linear equations and statistical parameters were obtained, considering a Student's *t*-test (two-tailed) with *n* − 2 degrees of freedom (Table 2). Considering an invalid correlation between AOF concentration and measured absorbance (Null hypothesis H_0_), the results gave *t*_calculated_ > *t*_critic_ for each time value, causing the hypothesis refusal and confirming the linear behavior for each time value. The linear and statistical parameters, for *t* = 30 min, were optimal. Since this method shows a marked increase in sensitivity it was selected for the determination of AOF.

### 4.6. Calibration Curve and Analytical Parameters Using AOF Standards Solutions

Using the previously selected experimental conditions, the analytical parameters were obtained for drug standard solutions with 11 independent reagent blanks (without analyte) (Table 3).

The accuracy and precision were evaluated for three AOF concentrations (8.00 × 10^−6^; 10.0 × 10^−6^; 30.0 × 10^−6^ mol L^−1^), through the recoveries and Student's *t*-test, respectively. Recoveries for intraday and interday (Table 4) were obtained, with 30 blanks in 5 consecutive days of measurement, with Student's *t*-test of 95% confidence percentage and *n* − 1 degrees of freedom. The results gave *t*_calculated_ < *t*_critic_, indicating that the differences between observed and expected values are acceptable given the confidence percentage established as criterion of acceptability. These results indicated satisfactory repeatability and precision. The recoveries are between 101.3 and 106.5%, presenting a satisfactory accuracy for kinetic-analytical methods evaluated.

### 4.7. Validation and Application

The validation of the above procedure was carried out in tap water samples fortified with three levels of AOF concentration (8.00 × 10^−6^; 10.0 × 10^−6^; 30.0 × 10^−6^ mol L^−1^) following the procedures of Section 3.3. For this, a calibration curve was prepared in tap water (2.3 × 10^−6^–3.2 × 10^−5^ mol L^−1^) using the respective blanks (without AOF). With these results, the analytical parameters indicated in the Table 5 were obtained.

Afterwards, intraday and interday assays of a group of 30 blanks were realized; measurements were carried out for five consecutive days. With these results, the standard deviation was determined (Table 6). In order to assess the accuracy of the method, recoveries were obtained using three analyte concentrations, mentioned above. The recoveries of the intraday and interday assays were 94.0–102.4% and 101.0–105.0% presenting a satisfactory accuracy for kinetic-analytical method selected (Table 6). The effect of the possible interfering was eliminated through the application of the procedure of Section 3.3.

The method was applied in spiked tap water samples with three known amounts of AOF and then analyzed individually (6 repetitions) according to the respective procedure. The linear regression equation obtained in Section 4.7 was used in the AOF quantitation. For the proposed samples the statistical analysis of these results using Student's *t*-test showed that there was no significant difference between the real and found concentrations at the 95% confidence level (Table 6, intraday).

## 5. Conclusions

In this study a kinetic spectrophotometric method based on the AOF oxidation with KMnO_4_ in alkaline medium to form to MnO_4_^2−^ was carried out. Optimal results were obtained by measuring the oxidation kinetic of AOF-KMnO_4_ system using 9.0 × 10^−4^ mol L^−1^ KMnO_4_, 50°C, 0.20 mol L^−1^ Na_2_SO_4_, and 0.34 mol L^−1^ NaOH for 30 min. The molar relation AOF : KMnO_4_ was determined using the previously stated variable values and was 1 : 1.

The kinetics studies were carried out considering three methods: initial rate, constant rate, and fixed time. The most suitable method was the fixed time at 30 min, because it presented satisfactory values of analytical parameters (*R*^*2*^ and slope). To assay the accuracy and reliability of the AOF determination in the proposed sample several assays were carried out and showed statistically satisfactory results with the recoveries and the respective Student's *t*-test. This method is simple and does not require expensive instruments and complicated extraction steps of the reaction product.

## Figures and Tables

**Table 1:** Coded values for the factorial design.

Factor	−1	0	+1
KMnO_4_ (×10^−4 ^mol^ ^L^−1^)	7	8	9
Na_2_SO_4_ (mol^ ^L^−1^)	0.2	0.3	0.4
NaOH (mol^ ^L^−1^)	0.018	0.09	0.18
Temperature (°C)	35	50	65

**Table 2:** Calibration curves and statistical parameters for different fixed times at 50°C, monitoring at 610 nm and AOF concentration ranging between 6.0 × 10^−6^ and 3.0 × 10^−5 ^mol L^−1^.

Parameters	0 min	10 min	20 min	30 min	40 min	50 min	60 min
*R* ^2^	0.9899	0.8744	0.9161	0.9544	0.9345	0.9426	0.9619
Typical error	0.00462	0.0587	0.0595	0.0538	0.0812	0.0617	0.0490
*t*_calculated_	17.15	4.570	5.726	7.926	5.114	7.019	8.709
Intercept	0.00250	0.0461	0.0562	0.0472	0.0838	0.1124	0.1550
Slope (×10^3^)	2.244	7.600	9.655	12.09	11.77	12.27	12.10

*t*_critic(*P*=0,05,*n*−2)_ = 3.18, *N* = 5.

**Table 3:** Analytical parameters using AOF standards solutions.

Parameters	Values
Linear regression	*A* = 1.364 × 10^4^*C* + 5.20 × 10^−3^
*R* ^2^	0.997
LOD^*∗*^ (mol L^−1^)	2.39 × 10^−6^
LOQ^*∗*^ (mol L^−1^)	7.25 × 10^−6^
Linear range (mol L^−1^)	7.25 × 10^−6^–3.22 × 10^−5^
*σ* (blanks)	9.90 × 10^−3^

^*∗*^LOD = 3.3*σ*/S and LOQ = 10*σ*/S.

**Table 4:** Analysis to assess the precision and accuracy of the proceeding developed for the determination of AOF intraday and interday.

Added (×10^−6 ^mol L^−1^)	Found (×10^−6 ^mol L^−1^)	*σ* × 10^−7^	ES^*∗*^ × 10^−7^	Confidence limits^†^ × 10^−7^	*t*_cal_^*҂*^	Recovery (%)
Intraday						
8.00	8.10	2.68	1.55	6.66	0.219	101.3
10.0	10.6	106	69.9	3.00	3.09	106.5
30.0	32.2	322	1.18	5.08	3.49	103.9

Interday						
8.00	8.40	6.76	3.90	0.0167	3.42	105.0
10.0	10.5	6.97	4.02	0.0173	4.14	105.0
30.0	32.2	6.10	3.52	0.0151	11.4	103.9

^*∗*^Error standard deviation, ^†^confidence limits (mol L^−1^) with 95% and 5 degrees of freedom for intraday and for interday assays (*t*_critic_ = 4.3), and ^**҂**^*t*_cal_ = *t*_calculated_.

**Table 5:** Analytical parameters for the AOF determination using a calibration curve in tap water.

Parameters	Values
Linear regression	*A* = 1.31 × 10^4^*C* + 0.0111
*R* ^2^	0.999
LOD^*∗*^ (mol L^−1^)	2.49 × 10^−6^
LOQ^*∗*^ (mol L^−1^)	7.56 × 10^−6^
Concentration range (mol L^−1^)	7.56 × 10^−6^–3.22 × 10^−5^
*σ* (blanks)	9.90 × 10^−3^

^*∗*^LOD: 3.3*σ*/S; LOQ: 10*σ*/S.

**Table 6:** Analysis to assess precision and accuracy of the developed method for the determination of AOF intraday and interday.

Added (×10^−6 ^mol L^−1^)	Found (×10^−6 ^mol L^−1^)	*σ* × 10^−7^	ES^*∗*^ × 10^−7^	Confidence limits^†^ × 10^−7^	*t*_cal_^*҂*^	Recovery (%)
Intraday						
8.00	7.52	5.86	2.39	6.15	0.469	94.0
10.0	10.2	4.68	1.91	4.92	0.291	102.4
30.0	30.2	2.70	1.10	2.84	0.514	100.8

Interday						
8.00	8.23	6.13	3.54	1.52	0.216	102.9
10.0	10.5	11.5	6.65	2.86	0.257	105.1
30.0	30.3	12.6	7.25	3.12	0.142	101.0

^*∗*^Error standard deviation, ^†^confidence limits (mol L^−1^) with 95% and 5 degrees of freedom for intraday and for interday assays (*t*_critic_ = 4.3); ^**҂**^*t*_cal_ = *t*_calculated_.
